# Prosocial Engagement Reduces Loneliness Among Older Adults Through: The HEAL-HOA Dual Randomized Controlled Trial

**DOI:** 10.1093/geroni/igaf122.1880

**Published:** 2025-12-31

**Authors:** Da Jiang, Dannii Yeung, Lisa Warner, Namkee Choi, Rainbow Tin, Hung Ho, Jojo Yan, Yan Kwok, Kee-Lee Chou

**Affiliations:** The Education University of Hong Kong, Hong Kong, China (People’s Republic); City University of Hong Kong, Hong Kong, China (People’s Republic); MSB Medical School Berlin, Berlin, Germany; The University of Texas at Austin, Austin, Texas, United States; The University of Hong Kong, Hong Kong, China (People’s Republic); The University of Hong Kong, Hong Kong, China (People’s Republic); The Education University of Hong Kong, Hong Kong, China (People’s Republic)

## Abstract

The COVID-19 pandemic has exacerbated loneliness among older adults, highlighting the critical need for targeted interventions to reduce loneliness among older adults. Adopting an “peer-support framework”, we conducted a dual randomized controlled trial (HEAL-HOA RCT) to evaluate the effects of structured volunteering on reducing loneliness in older adults. Participants aged 50–70 (N = 375) were randomized into either volunteering or a psychoeducation group. Volunteers received training and then delivered one of three telephone-based psychosocial interventions (mindfulness, behavioral activation, or befriending) to lonely seniors aged 65+ who were living alone, below the poverty line and without internet. Volunteers committed to intervention delivery 2 hours/week for six months. Primary and secondary outcomes were assessed at baseline, 6 months (T2), and 12 months (T3). Results revealed significant loneliness reductions among volunteers at T2 (medium/large effect sizes) measured by the UCLA Loneliness Scale and De Jong Gierveld Loneliness Scale. Sustained benefits at T3 were only observed in the participants who maintained >2 hours/week of volunteering. Volunteering engagement also improved secondary outcomes, including (Lubben Social Network Scale & Multidimensional Scale of Perceived Social Support), life satisfaction (Satisfaction with Life Scale), and psychological well-being (Psychological Wellbeing Scale) at T2. These findings underscore the dual value of prosocial acts: addressing community needs while improving older adults’ own mental health. Structured, sustained volunteering programs are essential, as benefits depend on continued engagement beyond initial commitments. This study bridges gaps in understanding causal effects of volunteering and offers actionable strategies to promote healthy aging through purposeful engagement.

